# Clinical equivalence of Trusynth fast ^®^ and Vicryl rapide ^® ^polyglactin 910 fast absorbing sutures on maternal morbidity experienced by women following episiotomy repair: a single-blind, randomized study

**DOI:** 10.12688/f1000research.126555.2

**Published:** 2023-06-23

**Authors:** Dongabanti Hemalatha Devi, Chethana Bolanthakodi, Prema D’Cunha, Mudiki Bheema Bai, Ashok Kumar Moharana, Deepak TS

**Affiliations:** 1 Obstetrics & Gynecology, Government Victoria Hospital, Visakhapatnam, Andhra Pradesh, 530001, India; 2 Obstetrics & Gynecology, Father Muller Medical College, Mangalore, Karnataka, 575002, India; 3 Clinical Affairs, Healthium Medtech Limited, Bangalore, Karnataka, 560058, India

**Keywords:** Episiotomy healing scale, Episiotomy repair, Perineal pain, Polyglactin 910 fast absorbable suture, Vaginal delivery, Wound complications

## Abstract

**Background: **Episiotomy procedure enlarges the vaginal outlet to facilitate childbirth. Polyglactin 910 fast-absorbing sutures are widely used for the repair of episiotomy because of their rapid absorption and less inflammatory response. This study was designed for subjective assessment of perineal pain post-episiotomy repair, with Trusynth Fast ^®^ and Vicryl Rapide ^®^ polyglactin 910 fast-absorbing sutures.

**Method**: This was a single-blind, randomized, prospective study conducted between January 7, 2021 and July 14, 2021 across two centers in India. Primiparous or multiparous women (18—40 years), who required episiotomy during vaginal delivery were included, and either Trusynth Fast ^® ^(n=47) or Vicryl Rapide ^® ^(n=49) suture was used for their episiotomy repair. The primary endpoint, perineal pain was assessed with visual analogue scale at all follow-up visits. The secondary endpoints, quantity of local anesthesia, number of sutures used, time to repair episiotomy, intraoperative suture handling, analgesics used, early and late wound complications, wound re-suturing, time to complete healing, presence of residual sutures, return to sexual activity, dyspareunia, and adverse events were also recorded.

**Results**: The study showed no significant difference in perineal pain between the two groups at any visit. A statistically significant difference (p<0.05) in total score of episiotomy healing scale on day 2 (0.13±0.34 *versus* 0.35±0.56) and swelling on day 2 (8.51 *versusversus* 28.57%) was noted between Trusynth Fast ^®^ and Vicryl Rapide ^®^ group. Non-significant difference was observed between the groups regarding anesthesia, number of sutures, time to repair episiotomy, intraoperative suture handling, analgesics, puerperal fever, wound infection, dehiscence, hematoma, urinary incontinence, re-suturing, time to complete healing, return to sexual activity and dyspareunia.

**Conclusion**: Trusynth Fast ^®^ suture is clinically equivalent to Vicryl Rapide ^®^ suture and can be used for episiotomy repair with minimal risk of perineal pain and wound complications.

Clinical Trials Registry of India Registration: CTRI/2020/12/029925; Registered on December 18, 2020

## Introduction

Episiotomy, the most frequently performed operative procedure enlarges the vaginal outlet in order to facilitate childbirth. It is performed by applying an incision on the perineum. In India, episiotomy is performed in approximately 63% of women during vaginal delivery. ^1^ Generally, episiotomy is associated with wound infection, wound dehiscence, perineal pain and discomfort. ^2^ About 46% of mothers in lower income countries experience some degree of trauma to the perineal area during vaginal delivery. ^3^

After performing episiotomy, the incised tissue has to be approximated, which is significantly impacted by the suture material used for tissue approximation, as it may affect perineal pain, discomfort and episiotomy healing. ^4^ Although chromic catgut suture material was most commonly used for episiotomy repair, a higher incidence of short-term morbidity was found after using it in comparison to absorbable synthetic material. ^5^ Furthermore, in comparison to chromic catgut sutures, use of polyglactin 910 sutures reported lower cases of morbidity and perineal pain. ^6^ The fast-absorbing polyglactin 910 sutures accelerate hydrolysis and take 42 days to be absorbed. ^7^ Previous studies compared synthetic absorbable suture materials (both polyglactin-910 and fast-absorbing polyglactin 910) with catgut sutures for episiotomy repair, ^5^^,^^8^^,^^9^ but comparison between Trusynth Fast ^®^ and Vicryl Rapide ^®^ fast-absorbing polyglactin 910 sutures for episiotomy repair has not been done yet. Therefore, this study was designed to compare Trusynth Fast ^®^ and Vicryl Rapide ^®^ fast-absorbing polyglactin 910 sutures for evaluating maternal morbidity experienced by women following episiotomy repair.

## Methods

### Study design

This was a single-blind, randomized, prospective study conducted between January 7, 2021 and July 14, 2021 across two centers in India. The primary objective was subjective assessment of perineal pain post-episiotomy repair, with Trusynth Fast ^®^ and Vicryl Rapide ^®^ suture. The secondary objectives included assessment of overall intraoperative handling, use of analgesics, wound healing, resumption of sexual activity and dyspareunia, number of sutures utilized and residual suture removal and maternal morbidities post-episiotomy in Trusynth Fast ^®^ and Vicryl Rapide ^®^ suture groups.

### Ethical approval

This trial was registered at Clinical Trials Registry of India (CTRI Reg. No: CTRI/2020/12/029925; December 18, 2020) and the Institutional ethics committee of both participating sites approved the study protocol. Government Victoria Hospital-King George hospital ethics committee, approved the study on 4 ^th^ December 2020, and Father Muller Institutional ethics committee approved the study on 15 ^th^ December 2020 with registration number FMIEC/CCM/693/2020. The study is reported as per Consolidated Standards of Reporting Trials (CONSORT), and details regarding the CONSORT check list is present in data availability section.

### Informed consent

Written informed consent was obtained from all participants for participation in the study as well as for publication of their clinical data.

### Study participants

Primiparous or multiparous women aged 18 to 40 years, with a singleton pregnancy, gestational age of >34 weeks, and good systemic/mental health, who required episiotomy during the course of vaginal delivery, who visited the Department of Obstetrics & Gynecology of both the centers were invited to participate in this research. They were included in this study after obtaining informed consent.

Women were excluded if they had intrapartum fever, tears (perineal, cervical or vaginal) or extension of the episiotomies, HIV or hepatitis B infection, stillbirth, known allergy to the suture materials and a history of bleeding/coagulation disorders or perineal surgery other than the primary repair after childbirth.

The women were also excluded if the investigator felt it would be difficult to follow the study procedure and follow-up.

### Study settings

The study was conducted at two sites: (i) Department of Obstetrics & Gynecology, Government Victoria Hospital, Visakhapatnam-530001, Andhra Pradesh, India, and (ii) Department of Obstetrics & Gynecology, Father Muller Medical College, Mangalore-575002, Karnataka, India.

### Intervention

The two studied interventions were Trusynth Fast ^®^ (Healthium Medtech Limited) and Vicryl Rapide ^®^ (Ethicon, Johnson and Johnson) sutures. Both sutures are coated, braided, absorbable sterile polyglactin 910 fast absorbable sutures and indicated for use in soft tissue approximation, where only short-term wound support is required, and rapid absorption of the suture can be beneficial.

### Study procedure

On the day of delivery (Baseline visit or day 0), the episiotomies were repaired as per standard institutional protocol with either Trusynth Fast ^®^ or Vicryl Rapide ^®^ sutures. The time between giving of the episiotomy and the time of start and completion of suturing was noted. The subjects were followed on day 2 (In-patient visit while in the hospital), day 11 (In-clinic/Telephonic visit), week 6 (In-clinic visit) and week 12 (Telephonic visit).

### Demographics

Baseline demographics including age, ethnicity, smoking and alcohol consumption history, weight, height, vital signs along with period of gestation, parity, history of previous episiotomy, fetal presentation in utero and medical/surgical history were recorded.

### Study outcomes

***Primary outcome***

The primary outcome, perineal pain following repair of episiotomy at 2 hours, 4 hours, 6 hours, and 12 hours after surgery, and on the day 2, day 11, week 6, and week 12 were noted using the visual analogue scale (VAS). VAS of 0–4 was graded as no pain, 5–44 as mild pain, 45–74 as moderate pain and 75–100 as severe pain.

***Secondary outcome***

The secondary endpoints, quantity of local anesthesia, number of sutures used, time to repair episiotomy, intraoperative suture handling, post-episiotomy number and dosage of analgesics, early and late wound complications, *viz.* puerperal fever (fever caused by uterine infection), swelling, infection (mild to severe discharge requiring treatment), hematoma (wound swelling >1 cm with changing color of skin), wound gaping, disruption or dehiscence (separation of wound edges of ≥1 cm) and urinary incontinence (involuntary loss of urine with coughing, sneezing, laughing, or running), time to complete healing, presence of residual sutures, frequency of wound re-suturing, return to sexual activity, dyspareunia and adverse events were evaluated.

The intraoperative suture handling was assessed using parameters like ease of passage through tissue, first-throw knot holding, knot tie-down smoothness, knot security, stretch capacity, memory, suture fraying on a five-point scale: 1 poor; 2 fair; 3 good; 4 very good; and 5 excellent. Wound healing was assessed by the standardized and valid REEDA (redness, edema, ecchymosis, discharge, and approximation) scoring scale, with scores ranging from 0 to 15. A lower score indicates better healing at the episiotomy site and higher score shows poor healing processes. Any unanticipated clinical signs, medical condition, disease or injury during the study period, which were already captured as study endpoints were not labeled and reported as adverse events.

Other standard details about the duration of second stage of labor, length of incision, number of layers closed, suture related challenges, perioperative complications, postpartum hemorrhage, outcome of surgery, length of hospital stay, antibiotic prophylaxis, suture loosening, feeling of slight stitches, and suture sent for culture were also recorded. In addition, the prescribed/concomitant medications during the study period were also noted.

### Sample size

The data from a previous study found that one out of 50 (2%) women in Vicryl Rapide ^®^ group had mild discomfort in sitting posture at six weeks, and 98% had no discomfort or pain. ^9^ Following the findings of this study, the proportion of patients experiencing no perineal pain, post-episiotomy repair, in the standard Vicryl Rapide ^®^ arm was assumed to be 98% i.e., π _1_=98%. Assuming type I error as 5% (α=0.05), power as 80% (1-β=0.8) and a difference to be detected as 1% for the proportion of patients experiencing no perineal pain in the Trusynth Fast ^®^ arm (π _2_=97%) with a margin of non-inferiority as 10% of the difference (δ), a minimum sample size was determined approximately as 38 in each arm. Further, considering a drop out of 20% and post-randomization exclusion of 10%, the required sample size was increased to 50 in each arm. So, a total of 100 subjects participated in this trial.

Sample size calculation formula:

$$\text{Two-sample Parallel}\;\text{Non-inferiority}\;\pi_{1}-\pi_{2}\geq \delta \;n_{i}=\frac{{\left(z_{\alpha }+z_{\beta }\right)}^{2}\left(\pi_{1}\left(1-\pi_{2}\right)+\pi_{2}\left(1-\pi_{2}\right)\right)}{{\left(\pi_{1}-\pi_{2}-\delta \right)}^{2}}$$

n _i_: sample size required in each group; Z _α_: conventional multiplier for alpha; Z _β_: conventional multiplier for power; π _1_: proportion of patients experiencing no perineal pain in the Vicryl Rapide ^®^ arm; π _2_: proportion of patients experiencing no perineal pain in the Trusynth Fast ^®^ arm; δ: margin of non-inferiority difference; π _1_-π _2_: size of difference of clinical importance.

### Randomization and blinding

Before initiation of the study, a computer-based, automated randomization number was generated by using version 1.0 of Random Allocation Software, using block sizes of 4, 6 or 8 by an independent programmer. The randomization concealment was done by sequentially numbered opaque sealed envelopes (SNOSE) technique. The subjects were allocated randomly in a 1:1 ratio to Trusynth Fast ^®^ (n=50) or Vicryl Rapide ^®^ (n=50) suture group. This was a single-blind study and the subjects were kept blinded to the device allocation status.

### Statistical analysis

The per-protocol or PP analysis set was used for statistical analysis using the SPSS version 25.0 (SPSS, Chicago, Illinois, USA). The PP set includes all subjects, who have complete data on the primary effectiveness parameter during 12 weeks follow-up. All continuous variables were expressed as mean±SD (standard deviation) and compared using the t-test for normally distributed data or Mann-Whitney U test for distribution-free data. All qualitative variables were expressed as proportions/percentages, and compared using Chi-squared test or Fisher's Exact test. A p value of < 0.05 was considered statistically significant.

## Results

**Figure 1.**
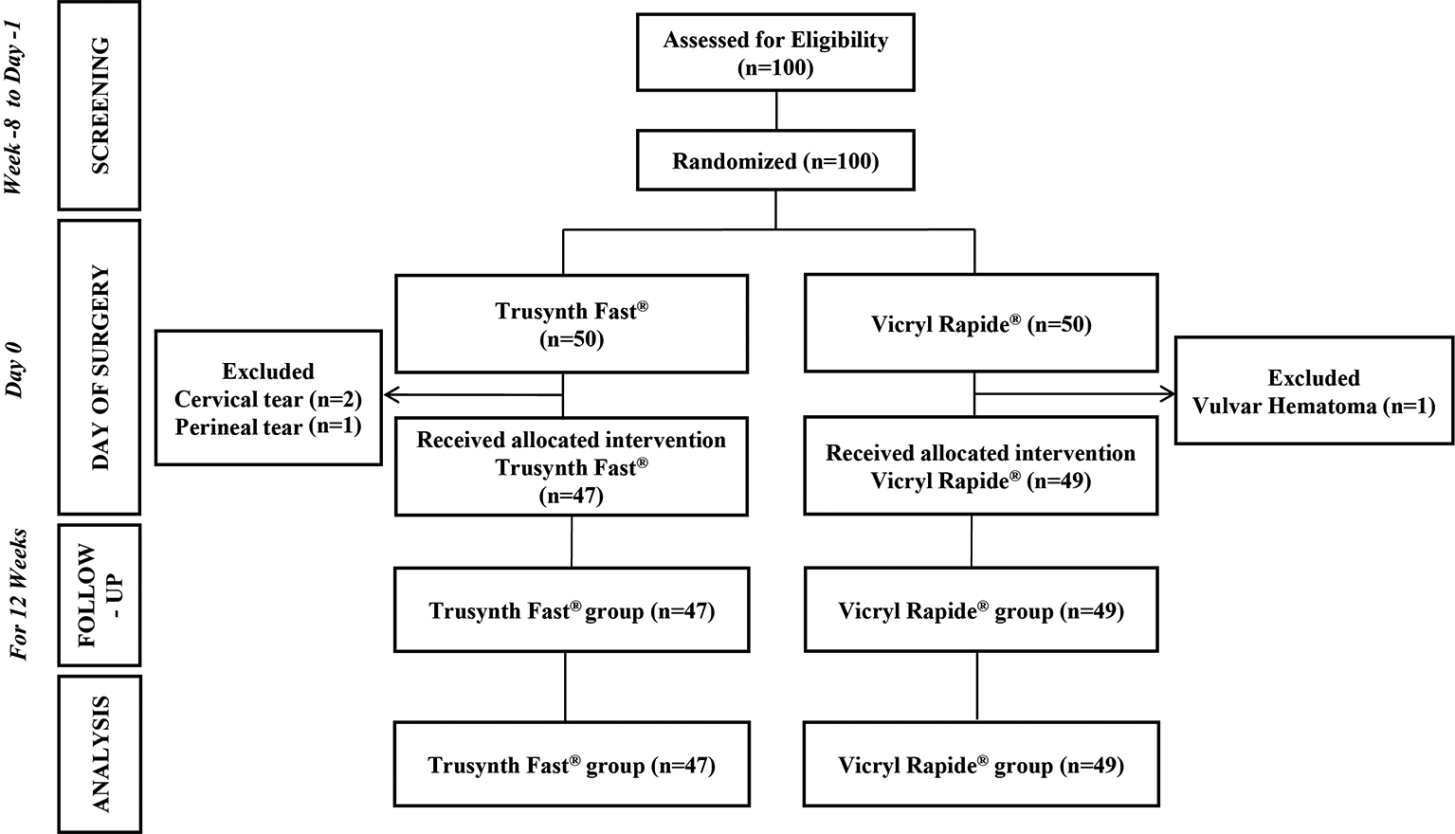
CONSORT flow diagram of the study participants and the study design.

A total of 100 women were screened for eligibility between January 7, 2021 and April 21, 2021 and follow-up of the last subject was completed on July 14, 2021. However, four subjects met the exclusion criteria and did not receive the intervention. Therefore, the PP set consisted of 96 subjects, who received the allocated intervention (Trusynth Fast ^®^, n=47; Vicryl Rapide ^®^, n=49) for episiotomy repair and completed the study ( Figure 1).

### Baseline demographics

Baseline demographics, vital signs and other relevant characteristics were comparable between the two treatment arms ( Table 1). All the subjects who participated in the study were Indians and none of them had a history of alcohol consumption or smoking. The fetus of all the studied women was in vertex position.

**Table 1.** Baseline demographics and other relevant characteristics of the study participants.

Parameters	Trusynth Fast® (n=47)	Vicryl Rapide® (n=49)	p-value
Age (years), Mean±SD	25.54±4.29	25.46±4.55	0.63
Weight (kilogram), Mean±SD	61.36±10.77	59.39±8.66	0.16
Height (centimeter), Mean±SD	154.68±7.56	153.89±6.75	0.30
BMI (kilogram/meter ^2^), Mean±SD	25.58±3.57	25.11±3.34	0.86
Gestation period (weeks), Mean±SD	38.31±1.54	38.47±1.16	0.42
Parity number, n (%)			
*0*	20 (42.55)	24 ( 48.98)	0.88
*1*	23 (48.94)	20 (40.82)	
*2*	3 (6.38)	4 (8.16)	
*3*	1 (2.13)	1 (2.04)	
History of previous episiotomy, n (%)	19 (40.43)	18 (36.75)	0.71
Medical/surgical history, n (%)	8 (17.02)	11 (22.45)	0.45
Pulse rate (beats/minute), Mean±SD	82.66±6.23	83.12±4.42	0.68
Respiratory rate (breaths/minute), Mean±SD	17.11±1.77	16.80±1.59	0.37
Systolic blood pressure (mmHg), Mean±SD	118.30±12.04	119.18±11.15	0.71
Diastolic blood pressure (mmHg), Mean±SD	77.66±7.29	78.57±6.46	0.52

BMI=body mass index, SD=standard deviation, mmHg=millimeters of mercury.

### Primary endpoint analysis

**Figure 2.**
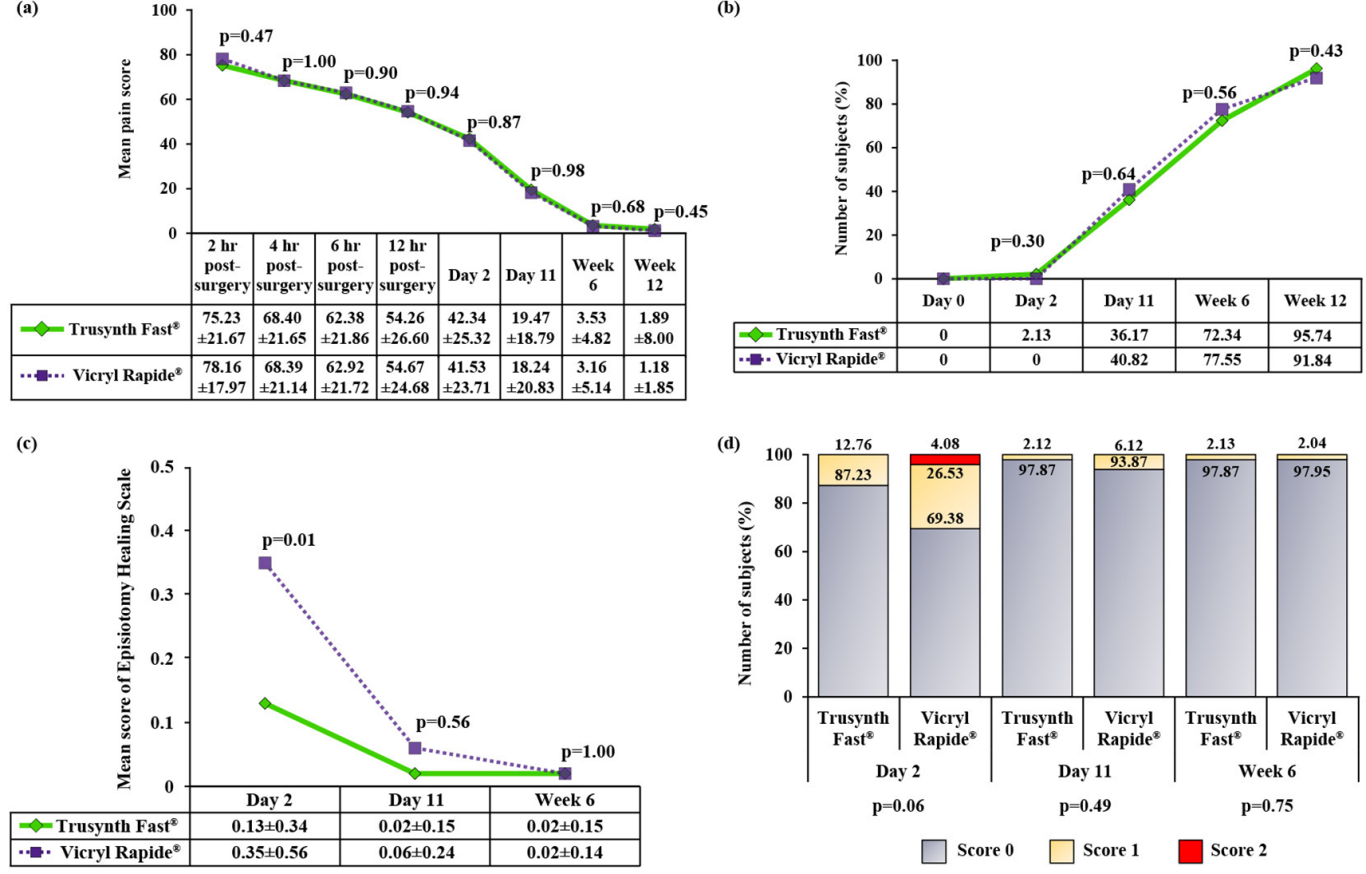
Post-operative profile of the subjects randomized to Trusynth Fast ^®^ (n=47) and Vicryl Rapide ^®^ (n=49) groups: (a) Mean pain score was evaluated using visual analogue scale (VAS) scale, (b) Frequency of subjects with no pain, (c) Mean total score of episiotomy healing scale, and (d) Percentage of score of Episiotomy Healing Scale or REEDA scale. hr: Hours, p<0.05 is statistically significant.

The perineal pain was assessed using VAS scale at 2 hours, 4 hours, 6 hours and 12 hours after childbirth and on all follow-up visits. No significant difference in perineal pain was observed, between the two groups at any time point. The intensity of pain was gradually decreased in subsequent follow-up visits ( Figure 2a and b).

### Secondary endpoint analysis

***Intraoperative profile***

**Figure 3.**
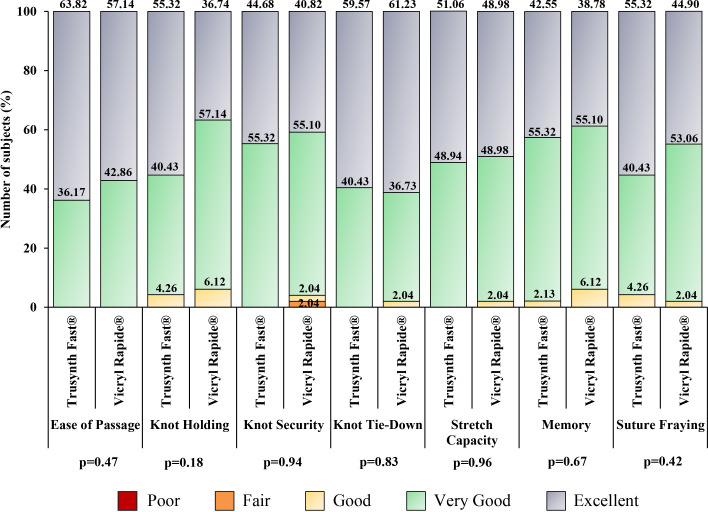
Intraoperative suture handling characteristics in subjects randomized to Trusynth Fast ^®^ (n=47) and Vicryl Rapide ^®^ (n=49) groups: Percentage scores for “excellent”, “very good”, “good” and “fair” are shown in one bar for different suture handling characteristics. None of the characteristics had “poor” score.

All subjects received 10 mL of local anesthesia (lignocaine) and antibiotic prophylaxis. In all subjects, normal delivery was done with no requirement for instrument use. All the episiotomies were right mediolateral incisions and three layers were closed in both groups. The results relative to the intraoperative handling characteristics are shown in Figure 3. “Excellent” score was higher for ease of passage, first-throw knot holding, knot security, stretch capacity, memory, and degree of fraying in Trusynth Fast ^®^ group, and for knot tie-down in Vicryl Rapide ^®^ group. Moreover, good outcome of surgery was noted in all subjects, as well as no suture-related challenges and perioperative complications were noted in both groups. The other intraoperative characteristics are summarized in Table 2.

**Table 2.** Intraoperative and post-operative profile of the study participants.

Parameters	Trusynth Fast ^®^ (n=47)	Vicryl Rapide ^®^ (n=49)	p-value
***Intraoperative profile***			
Duration of second stage of labor (hours), Mean±SD	0.70±0.43	0.68±0.47	0.86
Number of suture used, Mean±SD	1.43±0.54	1.49±0.51	0.58
Number of sutures used, n (%)			
*1*	28 (59.57)	25 (51.02)	0.29
*2*	18 (38.29)	24 (48.97)	
*3*	1 (2.13)	0	
Length of incision (centimeter), Mean±SD	4.30±0.83	4.14±0.68	0.30
Total time spent with repair of episiotomy (minutes), Mean±SD	15.26±4.43	13.98±3.85	0.13
Time of giving episiotomy to time of completion of suturing (minutes), Mean±SD	30.40±6.87	31.20±8.37	0.61
Mild atonic postpartum hemorrhage, n (%)	1 (2.13)	3 (6.12)	0.22
***Post-operative profile***			
Birth weight of infant (Kilogram), Mean±SD	2.92±0.29	2.83±0.44	0.24
Length of hospital stay (days), Mean±SD	4.15±1.14	3.92±1.10	0.32
Number of analgesics prescribed, Mean±SD			
*Day 0*	1.04 ± 0.29	1.04 ± 0.41	1.00
*Day 2*	0.98 ± 0.15	0.96 ± 0.20	0.58
*Day 11*	0.23 ± 0.43	0.33 ± 0.47	0.41
*Week 6*	0	0	-
*Week 12*	0	0	-
Swelling, n (%)			
*Day 2*	4 (8.51)	14 (28.57)	**0.01** *
*Day 11*	1 (2.13)	1 (2.04)	0.98
*Week 6*	0	0	-
*Week 12*	0	0	-
Time to complete healing (days), Mean±SD	3.19±3.18	6.96±11.12	0.18

SD=standard deviation.

* p<0.05.

***Post-operative profile***

The subjects of Vicryl Rapide ^®^ group took comparatively longer time for complete healing after episiotomy repair than Trusynth Fast ^®^ group, but the difference was not statistically significant ( Table 2). None of the subjects in any group required analgesic during week 6 and week 12 of delivery. A significantly higher (p<0.05) mean score of total episiotomy healing scale at day 2 in Vicryl Rapide ^®^ group indicated good healing in Trusynth Fast ^®^ group ( Figure 2c). At day 2, more subjects with score 0 for REEDA scale were found in Trusynth Fast ^®^ group, compared to Vicryl Rapide ^®^ group ( Figure 2d). The feeling of slight stitches was registered in both Trusynth Fast ^®^ and Vicryl Rapide ^®^ group at day 2 (mild, 2.13 *versus* 2.04%; moderate, 40.43 *versus* 40.82%, p=0.98), day 11 (mild, 31.92 *versus* 14.29%; moderate, 6.38 *versus* 8.16%, p=0.09) and week 6 (mild, 17.02 *versus* 14.29%, moderate 0 *versus* 0, p=0.71). At week 12, no subjects had the feeling of slight stitches.

None of the subjects were readmitted to the hospital during any follow-up visits. Incidence of suture loosening, residual suture removal and sent for culture were not recorded during entire study period. The incidence of swelling was observed in subjects of both Trusynth Fast ^®^ and Vicryl Rapide ^®^ groups at day 2 (8.51 *versus* 28.57%, p<0.05), that improved at day 11 (2.13 *versus* 2.04%, p=0.98), and became nil at week 6 and 12. No other wound complications (wound infection, wound dehiscence, hematoma, wound re-suturing, puerperal fever and urinary incontinence) were noted at any follow-up visits. After 6 weeks of delivery, 6 (12.77%) women in the Trusynth Fast ^®^ group and 6 (12.24%) women in the Vicryl Rapide ^®^ group were able to resume sexual activity (p=0.94). After 12 weeks of surgery, 21 (44.68%) women in Trusynth Fast ^®^ group and 17 (34.69%) women in the Vicryl Rapide ^®^ group resumed sexual activity (p=0.32). Furthermore, the women who resumed sexual activity did not report incidence of dyspareunia. Other post-operative characteristics are presented in Table 2.

Non-serious adverse events were observed, *viz.* drug allergy in one (2.13%) subject, mild vaginal discharge in one (2.13%) subject, and fever in 3 (6.38%) subjects of Trusynth Fast ^®^ group. In the Vicryl Rapide ^®^ group, one (2.04%) subject had general body pains along with vomiting and headache, one (2.04%) subject had fever, one (2.04%) subject had general body pains, and one (2.04%) subject had vomiting. The adverse events were mild in severity and not related to the study device. The prescribed/concomitant medications used during the study period are given in Table 3.

**Table 3.** Concomitant or prescribed medication during the study period.

Medications	Trusynth Fast ^®^ (n=47)	Vicryl Rapide ^®^ (n=49)
***Analgesics, n (%)***		
Diclofenac	32 (68.09)	29 (59.18)
Paracetamol	15 (31.91)	18 (36.73)
***Antibiotics, n (%)***		
Amoxicillin	42 (89.36)	42 (85.71)
Metronidazole	19 (40.43)	20 (40.82)

## Discussion

In India, more than 60% of women undergo episiotomy during vaginal delivery. ^1^ Post-episiotomy development of swelling, hemorrhage, hematoma, wound dehiscence and infection along with prolonged hospital stay, perineal pain and delayed resumption of sexual activity is already reported. ^2^ The repair technique and the type of suture material used have a major influence on the outcome of episiotomy repair. According to NICE guidelines, rapidly absorbable synthetic suture is the optimal material for perineal repair. ^10^ Existing literature demonstrated the beneficial effect of synthetic suture over chromic catgut suture material regarding post-episiotomy complications, perineal pain and discomfort. ^5^^,^^6^^,^^8^^,^^9^ However, the comparison of two polyglactin 910 fast absorbing suture brands for episiotomy repair post-vaginal delivery is still not established. Therefore, this study compared Trusynth Fast ^®^ and Vicryl Rapide ^®^ fast-absorbing polyglactin 910 sutures for evaluating maternal morbidity experienced by women following episiotomy repair.

Different aspects of intraoperative suture handling characteristics studied in this study were comparable between the suture groups. Ease of passage, knot security, knot tie-down and stretch capacity were graded as either “very good” or “excellent” in all subjects of Trusynth Fast ^®^ group, while only ease of passage was graded either “very good” or “excellent” in all subjects of Vicryl Rapide ^®^ group. None of the suture handling characteristics was graded “fair” or “poor” in Trusynth Fast ^®^ group, and “poor” in Vicryl Rapide ^®^ group. Furthermore, good outcome of surgery along with no suture related challenges indicates the clinical equivalence of both sutures.

Perineal pain for ≥72 hours of episiotomy is the common morbidity in women, for which analgesia can be continued for ≥10 days after delivery. ^2^ Use of a rapidly absorbable form of polyglactin 910 for the repair of perineal trauma offers a significant reduction in pain and analgesic number, when compared to standard absorbable synthetic material. ^11^ Polyglactin 910 elicits minimal tissue reaction as compared to catgut, and is associated with less pain. ^6^ A decrease in perineal pain and analgesic requirement with the use of a rapidly absorbable synthetic suture material was reported in previous studies. ^9^^,^^11^ Withstanding the findings of these studies, subjects of present study also showed reduction in perineal pain, from day 2 onwards after using Trusynth Fast ^®^ and Vicryl Rapide ^®^ fast-absorbing polyglactin 910 suture for episiotomy repair. In both suture arms, similar improvement in pain was evident, with no significant difference at any follow-up visit. In addition, the mean analgesics prescribed were also decreased with each passing visit in both groups, and not required by any subjects at 6–12 weeks.

Postpartum infection is a major cause of maternal mortality and also associated with maternal anxiety and postpartum depression. ^12^ Similarly, wound dehiscence is one of the important causes of re-suturing, and requires hospital re-admissions in the postpartum period, leading to physical and psychological problems. ^13^ The results of the present study depicted no wound infection, wound dehiscence, hematoma, puerperal fever or urinary incontinence. Moreover, re-suturing was not required in any of the subjects. However, a significantly higher incidence of swelling was observed in the Vicryl Rapide ^®^ group on day 2, which decreased eventually (within 11 days post-delivery), and no significant differences were found in the subsequent visits. A previous study reported that routine episiotomy resulted in inflammation along with hematoma, infection and dehiscence, pain, extension of the episiotomy incision, and sexual dysfunction. ^14^ However, in this study, comparable number of subjects in both groups resumed sexual activity by week 6 and 12, with no complaint of dyspareunia.

Perineal wound heals by primary closure, with least possible complications within 14 days of suturing. ^11^ Episiotomy repair with polyglactin 910 suture results in more satisfactory wound healing as compared to chromic catgut suture. ^8^ A non-significant, but faster wound healing was noted in Trusynth Fast ^®^ group as compared to Vicryl Rapide ^®^ group. In addition, no incidence of any suture-related adverse event was observed in this study.

This study is methodologically robust and appropriately powered to detect a difference in the primary and secondary outcomes; hence the findings of this study can be generalized to the wider population. The clinical equivalence of these two sutures regarding efficacy and safety indicates that Trusynth Fast ^®^ suture can be used in all surgeries indicated for Vicryl Rapide ^®^ suture. The limitation of the present study is that the surgeons who assessed the intraoperative suture handling characteristics were not blinded, and therefore, a potential bias might have occurred if they favored one suture over the other.

## Conclusion

The findings demonstrated clinical equivalence of Trusynth Fast ^®^ suture to Vicryl Rapide ^®^ suture in terms of non-significant differences in primary and secondary endpoints (except swelling and mean score of episiotomy healing scale on day 2). Both the sutures can be used for episiotomy repair with minimal risk for maternal morbidity, *viz.* perineal pain, early and late wound complications, and re-suturing.

## Data availability

### Underlying data

Figshare. Trusynth fast suture study Dataset, https://doi.org/10.6084/m9.figshare.21184411.v1. ^15^

This project contains the underlying data related to all the data points mentioned below:

• Demographic data, primary and secondary endpoints

Data are available under the terms of the Creative Commons Attribution 4.0 International license (CC-BY 4.0).

### Reporting guidelines

Figshare: Trusynth fast study CONSORT checklist, https://doi.org/10.6084/m9.figshare.21184420.v1. ^16^

Data are available under the terms of the Creative Commons Attribution 4.0 International license (CC-BY 4.0).
