# Supplementary material for: Clinical equivalence of Trusynth fast ® and Vicryl rapide ® polyglactin 910 fast absorbing sutures on maternal morbidity experienced by women following episiotomy repair: a single-blind, randomized study
Source: F1000Res. 2023 Jun 23;11:1179. Originally published 2022 Oct 17. [Version 2] doi: 10.12688/f1000research.126555.2 (PMC10300501; doi:10.12688/f1000research.126555.2)
Supplement: Peer review history

# Peer review: Reviewer response for version 2

**DOI:** 10.5256/f1000research.151757.r181232

**Published:** 2023-06-27

**Authors:** Shraddha Shetty K

**Affiliations:** 1 Department of Obstetrics and Gynecology, Kasturba Medical College, Manipal Academy of Higher Education, Mangaluru, Karnataka, India

No further comments to make. Article is approved.

Is the work clearly and accurately presented and does it cite the current literature?

Yes

If applicable, is the statistical analysis and its interpretation appropriate?

Yes

Are all the source data underlying the results available to ensure full reproducibility?

Yes

Is the study design appropriate and is the work technically sound?

Yes

Are the conclusions drawn adequately supported by the results?

Yes

Are sufficient details of methods and analysis provided to allow replication by others?

Yes

Reviewer Expertise:

High risk obstetrics and adolescent gynecology

I confirm that I have read this submission and believe that I have an appropriate level of expertise to confirm that it is of an acceptable scientific standard.

# Peer review: Reviewer response for version 2

**DOI:** 10.5256/f1000research.151757.r181233

**Published:** 2023-06-27

**Authors:** Geetika Kaur

**Affiliations:** 1 Wayne State University, Detroit, Michigan, USA

The article is scientifically valid in its current form.

Is the work clearly and accurately presented and does it cite the current literature?

Yes

If applicable, is the statistical analysis and its interpretation appropriate?

Partly

Are all the source data underlying the results available to ensure full reproducibility?

Yes

Is the study design appropriate and is the work technically sound?

Yes

Are the conclusions drawn adequately supported by the results?

Yes

Are sufficient details of methods and analysis provided to allow replication by others?

Yes

Reviewer Expertise:

Immunology, Cell and Molecular Biology, Angiogenesis, Pathology, Anatomy

I confirm that I have read this submission and believe that I have an appropriate level of expertise to confirm that it is of an acceptable scientific standard.

# Peer review: Reviewer response for version 1

**DOI:** 10.5256/f1000research.138979.r163358

**Published:** 2023-06-12

**Authors:** Shraddha Shetty K

**Affiliations:** 1 Department of Obstetrics and Gynecology, Kasturba Medical College, Manipal Academy of Higher Education, Mangaluru, Karnataka, India

**Brief description of the article:**

 This article aims at describing the type of suture material that can be used to repair the episiotomy. Benefits and complications of using Trusynth Fast and Vicryl Rapide polygactin 910 fast absorbing sutures. Primary outcome analysed was subjective post episiotomy perineal pain with the type of suture material used. Secondary outcomes analysed were quantity of local anesthesia, number of sutures used, time to repair episiotomy, intraoperative suture handling, analgesics used, early and late wound complications, wound re-suturing, time to complete healing, presence of residual sutures, return to sexual activity and dyspareunia. They concluded that Trusynth Fast® suture is clinically equivalent to Vicryl Rapide® suture and can be used for episiotomy repair with minimal risk of perineal pain and wound complications.

**Relevance: **Studying the type of suture material used for episiotomy and assessing the complications and side effects will help the clinicians in deciding the type of suture material to be used for episiotomy.

**Comments: **Dear authors, I read with great interest, which falls within the aim of this journal. In my honest opinion, the topic is of great interest to attract the readers’ attention.

**Major: **

- Aims and objectives have been mentioned clearly.
- Review of literature is appropriate for the study.
- Materials & Methods have been explained in brief.
- The study area has been defined clearly.
- Data collection technique is appropriate for the study.
- Data analysis technique is described in detail.
- Results have been described and analysed in detail.
- The discussion has been described clearly and results have been compared with other studies and analysed.

**Minor:**

- Introduction to the topic has been mentioned in detail.
- Conclusions and the summary of the study have been defined which is appropriate.

Is the work clearly and accurately presented and does it cite the current literature?

Yes

If applicable, is the statistical analysis and its interpretation appropriate?

Yes

Are all the source data underlying the results available to ensure full reproducibility?

Yes

Is the study design appropriate and is the work technically sound?

Yes

Are the conclusions drawn adequately supported by the results?

Yes

Are sufficient details of methods and analysis provided to allow replication by others?

Yes

Reviewer Expertise:

High risk obstetrics and adolescent gynecology

I confirm that I have read this submission and believe that I have an appropriate level of expertise to confirm that it is of an acceptable scientific standard.

# Peer review: Reviewer response for version 1

**DOI:** 10.5256/f1000research.138979.r171249

**Published:** 2023-06-02

**Authors:** Geetika Kaur

**Affiliations:** 1 Wayne State University, Detroit, Michigan, USA

The manuscript entitled “Clinical equivalence of Trusynth fast® and Vicryl rapide® polyglactin 910 fast absorbing sutures on maternal morbidity experienced by women following episiotomy repair: a single-blind, randomized study” by Devi *et al.* is quite interesting. The manuscript is well-written and provides additional evidence in the literature on the safe usage of polyglactin sutures. Detailed methodology and results have been provided. However, minor changes are required to improve the manuscript.

 Additional comments:

1. In the introduction section, provide characteristic features of Trusynth Fast sutures, if any previous studies have been conducted using it; they need not to be randomised but any general studies, suggesting the qualities of the suture.
2. In the methodology section, provide IEC no. of Government Victoria Hospital-King George hospital also, if any, similar to other institute’s registration number.
3. As mentioned in the result section, 100 females were screened and 4 were excluded. Were 100 consecutive women screened for eligibility? If yes, why were further patients not screened or recruited when four patients met exclusion criteria?
4. The study was conducted at two sites. How many patients were recruited from each site?
5. Figure numbers are not consecutive. Figure 1, 3 and then figure 2. Kindly rectify this.
6. In Figure 3, it would be better to mention SD along with mean values of pain score and mean healing score. It is not required to be mentioned for frequency of subjects.
7. In the post-operative profile of secondary endpoint analysis, the authors have mentioned that “The subjects of Vicryl Rapide® group took comparatively longer time for complete healing after episiotomy repair than Trusynth Fast® group, but the difference was not statistically significant (Table 2).”
  On applying two tailed t-test, the difference is found to be statistically significant with p value= 0.026.
  I would like to know which statistical test was applied to compare the mean values?
8. How many in clinic and telephonic follow up visits were conducted on Day 11, as mentioned in methodology? How did you verify the adverse events on telephonic follow-up?
9. In the discussion section, I would suggest to add a few studies from the literature to discuss the findings of this study.

Is the work clearly and accurately presented and does it cite the current literature?

Yes

If applicable, is the statistical analysis and its interpretation appropriate?

Partly

Are all the source data underlying the results available to ensure full reproducibility?

Yes

Is the study design appropriate and is the work technically sound?

Yes

Are the conclusions drawn adequately supported by the results?

Yes

Are sufficient details of methods and analysis provided to allow replication by others?

Yes

Reviewer Expertise:

Immunology, Cell and Molecular Biology, Angiogenesis, Pathology, Anatomy

I confirm that I have read this submission and believe that I have an appropriate level of expertise to confirm that it is of an acceptable scientific standard, however I have significant reservations, as outlined above.

# Response

**Published:** 2023-06-19

**Authors:** Chethana Bolanthakodi

**Affiliations:** Father Muller Medical College, India

The manuscript entitled “Clinical equivalence of Trusynth fast ^®^ and Vicryl rapide ^®^ polyglactin 910 fast absorbing sutures on maternal morbidity experienced by women following episiotomy repair: a single-blind, randomized study” by Devi *et al. *is quite interesting. The manuscript is well-written and provides additional evidence in the literature on the safe usage of polyglactin sutures. Detailed methodology and results have been provided. However, minor changes are required to improve the manuscript.

**Authors’ response: We appreciate your valuable time and efforts invested in reviewing this manuscript.**

**Additional comments**

 1. **Comment:** In the introduction section, provide characteristic features of Trusynth Fast sutures, if any previous studies have been conducted using it; they need not to be randomised but any general studies, suggesting the qualities of the suture.

**Response:** It is to bring to your attention that no studies are available in the literature on Trusynth Fast ^®^ suture. However, we have added studies related to fast-absorbing polyglactin 910 sutures in the introduction. [Page 4].

 Further, the characteristic features of both Trusynth Fast ^®^ and Vicryl Rapide ^® ^sutures are included in the Methodology section [Intervention; Page 6].

 2. **Comment: **In the methodology section, provide IEC no. of Government Victoria Hospital-King George hospital also, if any, similar to other institute’s registration number.

**Response:** No IEC number was provided on the ethical approval letter of King George Hospital. Therefore, it has not been mentioned in the manuscript.

 3. **Comment: **As mentioned in the result section, 100 females were screened and 4 were excluded. Were 100 consecutive women screened for eligibility? If yes, why were further patients not screened or recruited when four patients met exclusion criteria?

**Response:** Female patients were recruited on the basis of inclusion and exclusion criteria rather than consecutive recruitments. The four females were excluded after randomization and during surgery due to cervical tear, perineal tear, and vulvar hematoma, as shown in the CONSORT flow diagram.

 As mentioned in the sample size calculation, 76 females were to be included to attain significance. Thus, further recruitments were not conducted.

 4. **Comment: **The study was conducted at two sites. How many patients were recruited from each site?

**Response:** Both sites recruited an equal number of patients i.e., 50 females at each site.

 5. **Comment: **Figure numbers are not consecutive. Figure 1, 3, and then figure 2. Kindly rectify this.

**Response:** ​​​​​​​As per your suggestion, we have now rectified the same.

 6. **Comment: **In Figure 3, it would be better to mention SD along with mean values of pain score and mean healing score. It is not required to be mentioned for the frequency of subjects.

**Response:** ​​​​​​​We have now mentioned the same.

 7. **Comment: **In the post-operative profile of secondary endpoint analysis, the authors have mentioned that “The subjects of Vicryl Rapide® group took comparatively longer time for complete healing after episiotomy repair than Trusynth Fast® group, but the difference was not statistically significant (Table 2).”

**Response:** ​​​​​​​On applying a two-tailed t-test, the difference is found to be statistically significant with p value= 0.026.

 I would like to know which statistical test was applied to compare the mean values?

 Primarily, KS test was done to check the normality. The data was not found to be normally distributed thus, non-parametric approach, Mann-Whitney U test was used which resulted in p-value of 0.175. The difference was found to be statistically non-significant.

 8. **Comment: **How many in clinic and telephonic follow up visits were conducted on Day 11, as mentioned in methodology? How did you verify the adverse events on telephonic follow-up?

**Response:** ​​​​​​​Equivalent numbers of in-clinic and telephonic follow-ups were conducted on Day 11.

 During telephonic follow-up, the subjects were asked for the occurrence of any disease, injury, or any other signs for assessment of adverse events. In case of severe problems, the patients were asked for in-clinic visit.

 However, adverse events such as vaginal discharge, fever, general body pains, vomiting, and headache were reported.

 9. **Comment: **In the discussion section, I would suggest to add a few studies from the literature to discuss the findings of this study.

**Response:** ​​​​​​​Limited studies are available related to complications of episiotomy and post-episiotomy healing with polyglactin 910 sutures. We have included published articles as recent as possible to discuss the findings of the present study as described below:

- Improvement in perineal pain and a decrease in a number of analgesics with the use of polyglactin 910 suture has been discussed with respect to previous studies by Ononuju et al., 2020; Gupta et al., 2021; Samant et al., 2013 and Kettle et al., 2007 (Reference 2, 6, 9 and 11).
- Postpartum complications like infection, wound dehiscence and re-suturing, and requirement for hospital re-admissions have been discussed with respect to studies by Boushra and Rahman, 2013, and Dudley et al., 2017 (Reference 12 and 13). In addition, post-episiotomy inflammation, hematoma, infection and dehiscence, pain, extension of the episiotomy incision, and dyspareunia have been discussed after referencing the study of Gun et al., 2016  (Reference 14).
- Wound healing after episiotomy repair with Trusynth Fast ^®^ and Vicryl Rapide ^®^ group polyglactin 910 fast-absorbing sutures has been discussed with respect to studies by Howells and Abasi, 2020 and Kettle et al., 2007 (Reference 8 and 11).
